# Reevaluation of the Acute Cystitis Symptom Score, a Self-Reporting Questionnaire. Part I. Development, Diagnosis and Differential Diagnosis

**DOI:** 10.3390/antibiotics7010006

**Published:** 2018-01-15

**Authors:** Jakhongir F. Alidjanov, Kurt G. Naber, Ulugbek A. Abdufattaev, Adrian Pilatz, Florian M. E. Wagenlehner

**Affiliations:** 1 The Republican Specialized Center of Urology, 100109 Tashkent, Uzbekistan; jakhonghir@hotmail.com (J.F.A.); abdufattaev@gmail.com (U.A.A.); 2 Clinic of Urology, Paediatric Urology and Andrology, Justus-Liebig-University, 35392 Giessen, Germany; pilatz@t-online.de (A.P.); Florian.Wagenlehner@chiru.med.uni-giessen.de (F.M.E.W.); 3 Department of Urology, School of Medicine, Technical University of Munich, D-80333 Munich, Germany

**Keywords:** cystitis, female, quality of life, urinary tract infection, symptom score, questionnaire

## Abstract

This study aimed to reevaluate the Acute Cystitis Symptom Score (ACSS). The ACSS is a simple and standardized self-reporting questionnaire for the diagnosis of acute uncomplicated cystitis (AC) assessing typical and differential symptoms, quality of life, and possible changes after therapy in female patients with AC. This paper includes literature research, development and evaluation of the ACSS, an 18-item self-reporting questionnaire including (a) six questions about “typical” symptoms of AC, (b) four questions regarding differential diagnoses, (c) three questions on quality of life, and (d) five questions on additional conditions that may affect therapy. The ACSS was evaluated in 228 women (mean age 31.49 ± 11.71 years) in the Russian and Uzbek languages. Measurements of reliability, validity, predictive ability, and responsiveness were performed. Cronbach’s alpha for ACSS was 0.89, split-half reliability was 0.76 and 0.79 for first and second halves, and the correlation between them was 0.87. Mann-Whitney U test revealed a significant difference in scores of the “typical” symptoms between patients and controls (10.50 vs. 2.07, *p* < 0.001). The optimal threshold score was 6 points, with a 94% sensitivity and 90% specificity to predict AC. The “typical” symptom score decreased significantly when comparing before and after therapy (10.4 and 2.5, *p* < 0.001). The reevaluated Russian and Uzbek ACSS are accurate enough and can be recommended for clinical studies and practice for initial diagnosis and monitoring the process of the treatment of AC in women. Evaluation in German, UK English, and Hungarian languages was also performed and in other languages evaluation of the ACSS is in progress.

## 1. Introduction

Urinary tract infections (UTIs) are common, with an estimated annual global incidence of at least 250 million, with the vast majority (84%) of visits related to female patients presenting with acute uncomplicated cystitis (AC) [1,2,3]. Although AC is a benign condition, recurrent episodes are associated with a reduction in quality of life, a negative impact on everyday activity and working ability, disturbances in sexual life and psychosexual disorders [4,5,6]. In the recent years, several studies have shown specific risk factors for recurrent AC [7,8], and large studies revealed its microbial etiology. Clinical tests for diagnosing AC vary widely, depending on comorbidities and different treatment strategies [9,10,11].

Several studies and current guidelines do not consider urinalysis or microbiologic investigation necessary but rely on patients’ complaints only, even though telephone conversation [12,13,14]. Various urinary symptoms have been used to assess the diagnosis and severity of AC in women. Diaries, questionnaires, and symptom scores used in these studies were mainly adapted from other tools. Only a few publications devoted to the studies regarding the development of the questionnaires, evaluating the severity of symptoms of the AC and their interference with activities [15,16]. However, up to today, no single, unified, valid, and specific questionnaire existed for diagnosis, differential diagnosis, assessment of symptoms’ severity, and quality of life that is also available to monitor treatment efficacy [4,15,16,17]. We have recently presented a new self-reporting questionnaire—ACSS—for diagnosis, differential diagnosis, and patient-reported outcome in female patients with AUC, assessing typical and differential symptoms, quality of life, and possible changes after therapy [18,19].

Since the first 58 subjects who filled in the precursor version of the ACSS (USQOLAT) [20], which differed in a few aspects from the current version of the ACSS, were included in the former validation, a reevaluation of the current Uzbek and Russian versions of the ACSS was performed excluding these patients. Now, the present study includes only the results of the final 228 subjects (107 patients with AC and 121 controls without AC) who filled in the current versions of the Uzbek or Russian ACSS (Supplementary Figures S1 and S2).

## 2. Development and Evaluation Process of the Acute Cystitis Symptom Score (ACSS) Questionnaire

Regarding the purpose of the development of the ACSS questionnaire, PubMed’s medical subject headings (MeSH) service was used for the literature search. Different combinations of such MeSH terms as “urinary tract infections”, “cystitis”, and “signs and symptoms” were used for the identification of the key clinical symptoms/signs of AC. The literature search was limited to the publications in the English language. Thereafter, different versions of multiple choice questions regarding the presence and severity of symptoms were developed in Uzbek and Russian languages, and furtherly were revised and commented by Russian-speaking, Uzbek-speaking, and bilingual (speaking both in Uzbek and Russian languages) female staff of the hospital. Although the Uzbek language is the official state language in Uzbekistan, Russian is the second common language and remains in widespread use (https://en.wikipedia.org/wiki/Uzbekistan), with the majority of the population speaking both languages.

Following the approval of the Institutional Review Board (Clinical ethics committee) of the Republican Specialized Center of Urology/JSC “Republican Specialized Center of Urology” (RSCU) (Akilov FA, Mukhtarov ShT, Khvan AL: No. 429/23) in September 2009, preliminary versions of the questionnaire were tested as described earlier with females from different social areas, with different levels of education, nationalities, and age after informed consent was obtained prior to enrollment of the participants [18]. Based on their comments and requests, a multidimensional scaling analysis was performed, and the most appropriate versions of questions and answers were chosen by the research team for the development of the preliminary questionnaire titled as “Urinary Symptoms and Quality of Life Assessment Tool (USQOLAT).” The reliability analysis, performed on the first 58 subjects (32 patients with AC and 26 controls without AC), showed a mean Cronbach’s alpha of 0.84, a Split-half reliability of 0.82, and a Spearman-Brown prophecy of 0.9 [20].

From the preliminary (precursor) version (USQOLAT), the current ACSS was further updated as follows: (i) the scores for question 1 (frequency of urination) were related to the number of urinations per 24 h, (ii) the symptoms were clearly divided into “typical” and “differential” symptoms, and (iii) five questions on additional conditions were added.

## 3. Current Acute Cystitis Symptom Score Questionnaire (ACSS)

The current so-called ACSS is now an 18-item self-reporting questionnaire including (a) six items (questions) about “typical” symptoms of AC, (b) four items regarding differential diagnoses, (c) three items on quality of life, and (d) five items on additional conditions that may affect therapy (Supplementary Figures S1 and S2).

Part A of the ACSS is filled in at the first office visit, prior to treatment. Part A of the ACSS includes four subscales (domains): Typical, Differential, Quality of Life, and Additional. Part B of the questionnaire, which also includes the previously mentioned for domains of the Part A and “Dynamics” domain, in addition, is completed at a follow-up visit, e.g., after treatment [21].

## 4. Procedures for the Validation and Methods of the Statistical Analysis

### 4.1. Study Population

The Uzbek and Russian versions of the ACSS questionnaire (Supplementary Figures S1 and S2) were applied in 228 women (187 Uzbek and 41 Russian versions of the ACSS) presenting with and without symptoms of AC at the outpatient clinic of the RSCU. All participants signed written informed consent before filling out the questionnaire. Data obtained from filled-in paper-form questionnaires were then recorded into the electronic database, using PC software specially developed for the purpose of recording, storing and processing inputted data (e-USQOLAT). 

The clinical diagnosis of AC was established by the physicians of the RSCU based on subject’s complaints that are typical for UTI (acute onset, dysuria, urgency, the sensation of pain/discomfort above the pubic area, etc.) and the results of the laboratory tests. Respondents for whom the AC was ruled out were included in the control group, regardless of the total score obtained from the ACSS questionnaire or the presence or absence of other urological pathology.

### 4.2. Clinical Procedures

All patients were to undergo routine diagnostic procedures such as renal and bladder ultrasound, microscopy of centrifuged mid-stream urine sediment using a Goryaev’s chamber (hemocytometer) and urine culture. Urinalysis was performed according to Nechiporenko [22] with taken reference normal values of white blood cells (WBC) for females ≤2000/mL. Microscopic bacteriuria was registered as negative or positive. Any amount of bacteria was regarded as positive [23]. For the urine culture, a bacteriuria of ≥10^4^ colony forming units (CFU) per mL was considered positive. Other clinical and/or instrumental investigations and tests were performed when indicated. All required data, including patients’ answers, their age, ethnicity, employment status, place of residence, number of previous episodes of AC within the last year, duration of current episode, and results of laboratory tests, were recorded into the electronic database.

### 4.3. Validation Methods and Statistical Tests

#### 4.3.1. Testing Variables for Normality

The distributions of all the data obtained were tested visually (Q-Q Plots, histograms) and numerically (Shapiro-Wilk test). Parametric or non-parametric statistical tests were then performed in dependence of normality of distributions.

#### 4.3.2. Descriptive Statistics

Ordinary statistical parameters (mean, standard deviations, standard error etc.) were used for the description of patients’ demographics and characteristics.

#### 4.3.3. Validity and Reliability

Validity and reliability of the questionnaire were estimated calculating internal consistency by Cronbach’s alpha, split-half, and Spearman-Brown prophecy [24]. Spearman’s rank correlation coefficient was used for assessing relations between results of subjective self-assessment (answers marked on the questionnaire) and objective lower UTI symptoms (pyuria, hematuria, and bacteriuria).

#### 4.3.4. The Process of Distribution into the Groups

For revealing true negative and true positive, false-negative, and false-positive results, two members of the research team were chosen. One of them (JFA) had access to case histories and results of patient’s clinical and laboratory investigations but was blinded to the results of the questionnaire. The second member (UAA) was blinded to all results of patient’s investigations apart from the ACSS test results and final diagnosis of the urologist. Based on information available to them individually, they have made independent diagnostic decisions whether the respondent has AC or not. Their decisions were documented and then compared by the final decision maker (FAA, see “Acknowledgements”). In cases when their opinions coincided true-negative (both of them decided that the patient did not have AC) or true-positive (both of them decided that patient did have AC), diagnoses were marked. All disagreements were discussed with the project supervisor and final decision was achieved by consensus. Using this algorithm, patients were divided into two groups (patients with AC and controls without). 

#### 4.3.5. Predictive Ability and Responsiveness

Two-by-two (2 × 2) contingency tables were used for physician’s diagnosis taken as an outcome and binominal variable (positive/negative) taken as an exposure. To determine the predictive ability of the test, sensitivity and specificity were calculated. Odds ratios and absolute risk differences were also calculated.

Nonparametric Wilcoxon’s signed rank test was used for comparative analysis of variables for related samples, and parametric paired *t*-test was used for a reassessment of statistical significance [25,26].

The nonparametric Mann-Whitney test was used for comparing different variables between patients and controls. Wilcoxon signed rank test was used for comparing symptom severity before and after therapy in patients admitted for subsequent visits and assessing the responsiveness of the questionnaire.

Means and 95% confidence intervals (CI) were calculated. A *p*-value less than or equal to 0.05 was considered statistically significant. Substantive significance (effect size) was estimated by the modified correlation coefficient (r) proposed by Rosenthal and Rosnow using the Z-value retrieved from Wilcoxon’s signed-rank test.

The Statistical Package for the Social Sciences (IBM SPSS Statistics for Windows, Version 22.0. IBM Corp., Armonk, NY, USA) was used for statistical analysis and graphical presentations of the results.

## 5. Results

### 5.1. Development of ACSS Questionnaire

The literature search resulted in 56 articles covering the period from 1971 to 2012. Of those, 4 articles were selected as a basis for the items of the questionnaire [27,28,29,30]. The following main symptoms of women diagnosed with AC were identified: frequent voiding of small volumes, urgency, painful urination, feeling of incomplete bladder emptying after voiding, suprapubic discomfort and hematuria. These six symptoms were combined into the first domain of the questionnaire and labeled as “Typical”. Symptoms regarding other genitourinary problems (e.g., flank pain, vaginal/urethral discharge, fever, etc.) were selected for the second domain labeled as “Differential”. In the third domain, questions concerning the impact on quality of life were included and labeled as “Qol”. Finally, five questions regarding additional health conditions (menstruation, premenstrual and menopausal syndrome, pregnancy, diabetes mellitus) were labeled as “Additional”. For each question in the “Typical”, “Differential”, and “Qol” domains, a 4-point Likert-type scale assessing severity of each symptom ranged from 0 (no symptom) to 3 (severe) was introduced, while questions in the “Additional” domain contained only dichotomously fashioned “yes/no” questions. This procedure resulted in an 18 item self-reporting questionnaire for AC. 

### 5.2. Study Population: Characteristics of Patients and Controls

Among 228 women, 107 (46.9%), with mean age 30.02 ± 10.35 y.o., had physician-diagnosed AC. Of the 107 patients, urinalysis was performed for 106 (99.1%). Of those 106, 100 (94.3%) had pyuria (>2000 WBC/mL) and 75 (70.8%) had microscopic bacteriuria. Urine culture was performed for only 64 (59.8%) of all urine samples. Of them, 39/64 (60.9%) were culture-positive (CFU ≥ 10^4^/mL). The average duration of the episode at the time of the visit was 6.04 ± 4.37 days. All patients received appropriate treatment according to Uzbek national urological guidelines (standards), and a subsequent test-of-cure visit was recommended after its completion. Forty-eight of 107 patients (44.9%) came for a follow-up investigation after the treatment period and were asked to fill in the Part B of the questionnaire for further monitoring.

The control group consisted of 121 female patients (mean age 32.79 ± 12.69 years) without (*n* = 88) or with (*n* = 33) urologic pathologies (other than lower UTIs). There were no significant differences between patients and controls in terms of nationality, employment, age, pregnancy, and place of residence (*p* > 0.05).

### 5.3. ACSS Analysis in Subjects with AC and Controls at Visit 1

#### 5.3.1. Validity and Reliability

As the separately analyzed results of the Russian and Uzbek versions of the questionnaire published earlier did not differ significantly, a “combined” analysis for both versions was performed. Internal consistency and reliability of the ACSS were considered as good. Cronbach’s alpha for the entire questionnaire was 0.88, split-half reliability was 0.76 and 0.79 for both halves, Guttman split-half was 0.93, correlation (rho) between first and second halves was 0.87, and Spearman-Brown prophecy coefficient was 0.93.

Cronbach’s alpha for “Typical” domain (combination of the six most typical symptoms of AC) was 0.89, split-half reliability resulted in 0.82 and 0.76 for both halves, Guttman split-half was 0.92, and the correlation between first and second half was 0.85. Mean inter-item correlation of the domain was 0.55, the interclass correlation for average measures was 0.89 (95% CI: 0.87–0.91). 

The same tests performed for the “Differential” domain (five questions) showed the following results: Cronbach’s alpha 0.53, split-half reliability 0.35 and 0.21 for both halves, respectively, and Guttman split-half 0.57 and the interclass correlation coefficient was 0.53 (95% CI: 0.44–0.61).

The results of the reliability tests of the “QoL” domain (three questions regarding symptoms with negative influence on usual lifestyle) were excellent. In particular, Cronbach’s alpha was 0.91 average inter-item correlation was 0.76, the interclass correlation coefficient was 0.92 (95% CI: 0.90–0.93) and Spearman-Brown prophecy coefficient was 0.91.

Since items of the “Additional” domain had a dichotomous view, they were not included in this analysis.

#### 5.3.2. Discriminative Ability

**Figure 1:**
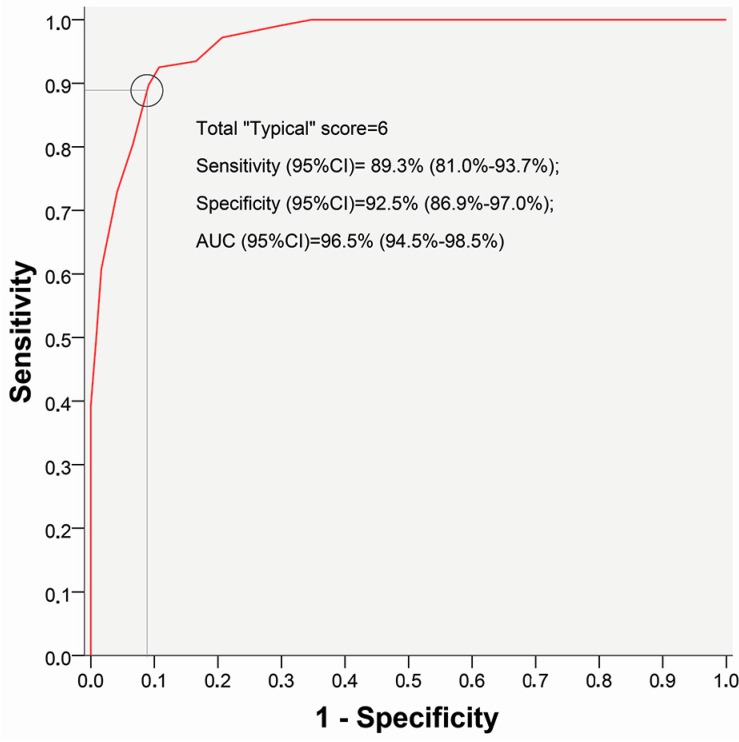
Receiver-Operating-Characteristic (ROC) curve analysis of the “Typical” domain of the Acute Cystitis Symptom Score (ACSS) at the first visit with results from 228 subjects (107 patients with acute uncomplicated cystitis (AC), 121 controls without AC).

**Figure 2:**
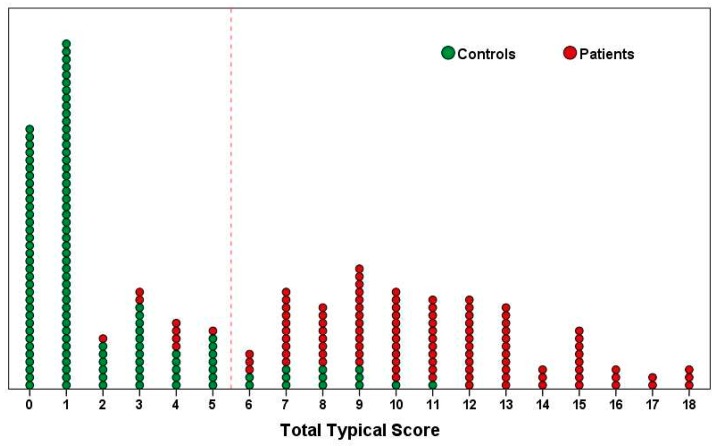
Distribution of 228 subjects (107 patients with AC, 121 controls without AC) according to a summary score of the “Typical” domain of the ACSS at the first visit.

Discriminative ability of the “Typical” domain was evaluated using Receiver-Operating-Characteristic (ROC) curve analysis (Figure 1). Changes in predictive ability of the questionnaire depending on scores in “Typical” domain showed that the most appropriate cut-off summary score for the “Typical” domain was equal to 6 (Table 1). Among 107 women with confirmed AC, 99 (92.5%) had a summary score of 6 and higher (true-positive result), and only 8 women (7.5%) with approved AC had a score less than 6 (false-negative result). Among the group of 121 women who did not have AC, the false-positive result was noted in 13 cases (10.7%) (false-positive value), and the true-negative result (<6) was reported for 108 women (89.3%). These results were similar to those published earlier, when the first 58 patients having filled in the precursor version, USQOLAT, were also included in the analysis (Table 2). A scatter plot chart according to a summary score of the “Typical” domain at visit 1 including 228 subjects (107 patients with AC, 121 controls without AC) is given in Figure 2.

#### 5.3.3. Predictive Ability and Responsiveness

Using a summary score of the “Typical” domain of 6 as discriminative, assessment of sensitivity and specificity resulted in values around 90% (92.5% and 89.3%, respectively) (Table 2). The positive likelihood ratio was as high as 8.6 and negative likelihood ratio was low enough (0.1). The absolute risk difference was 81.5%, and values of the odds ratio and relative risk for the AC group were 102.8 and 12.8, respectively (Table 3).

Differences in scores between the group of patients with AC compared to those without AC were significant (*p* < 0.0001) in items of “Typical” and “QoL” domains. Scores in items of the “Differential” domain did not differ significantly between groups except for questions regarding “vaginal discharge” and “urethral discharge” (Table 4).

For the group of patients at the time of the first visit, the most common symptom was urgency: 86 (80.37%) patients rated the intensity of this symptom as “moderate” and “severe” (46 and 40 patients, respectively). Painful urination was recognized as the second most common symptom, with 84 (78.50%) patients suffering correspondingly rating this symptom as moderate (43 patients) or severe (41 patients) (Table 5). 

Table 6 represents the distributions of scores of the typical symptoms in the groups of patients and controls. Note, blood in urine is only seen in patients with acute hemorrhagic cystitis, a specific form of cystitis, which is not the case in the majority of the patients with AC.

Table 7 shows total typical scores in patients and controls and in their corresponding subgroups: pregnant women, women ≤50 years of age and not pregnant, and women ≥51 years of age. The discrimination between patients and controls is in the subgroups as good as in the total group. Therefore, the ACSS is a reliable diagnostic tool for premenopausal, non-pregnant women, pregnant women, and postmenopausal women. 

### 5.4. ACSS Analysis in Subjects with AC and Controls at Follow-Up Visit

Of the total 107 patients with AC, 48 had subsequently a “test-of-cure” visit after 5.08 ± 2.71 days of therapy. Differences between scores obtained at the first and subsequent visits are shown in Table 8. The total “typical” symptom score decreased significantly when comparing the two visits (10.4 and 2.5, *p* < 0.001). A more detailed analysis will be discussed in part II [21].

## 6. Discussion

The consulting physicians diagnosed AC according to their clinical practice and based on the results of laboratory investigations. Besides the typical history and symptoms, urinalysis was performed in 99.1% patients (only 1 missing data) with positive pyuria in 94.5% of cases. Although microscopic bacteriuria and positive urine culture was only found in 75/106 (70.8%) and 39/64 (60.9%) of patients, respectively, it should be noted that only cases with a bacteriuria of at least 10^5^ CFU/mL found in microscopy [23] and at least 10^4^ CFU/mL in urine culture, could be considered as positive in the clinical practice.

In contrast, Stamm et al. had already shown that the traditional diagnostic criterion, ≥10^5^ CFU/mL of midstream urine, has a very high degree of diagnostic specificity (99%) but a very low level of sensitivity (51%) [31]. That means that this threshold of urine culture may identify only 51% of symptomatic women with lower UTI whose bladder urine (obtained by suprapubic aspiration or by catheter) contains coliforms. The authors found the best diagnostic criterion to be ≥10^2^ CFU/mL (sensitivity, 95%; specificity, 85%) and suggested that clinicians and microbiologists should alter their approach to the diagnosis and treatment of women with acute symptomatic coliform infection of the lower urinary tract.

Hooton et al., however, confirmed in a more recent study that colony counts of *E. coli* as low as even 10^1^ to 10^2^ CFU/mL in midstream urine were sensitive and specific for the presence of *E. coli* in catheter urine in symptomatic women [32]. Although using such low counts as a threshold, still no uropathogens could be cultured neither from catheter nor midstream urine in 22% of patients with typical symptoms of AC. It follows, that for diagnosing AC the symptomatology has become more decisive than any urinary bacterial count.

The results of our investigation demonstrate that the ACSS is a reliable, valid, and easy-to-use questionnaire, which may help to diagnose AC in women in primary healthcare settings and to assess treatment efficacy. The ACSS is a reliable diagnostic tool for patients and physicians, not only in premenopausal, non-pregnant women but also in pregnant women and in postmenopausal women. It can be self-administered and completed in a short time by respondents or surveyees. Questions and versions of answers are easy to understand and may be used for epidemiological surveys and/or drug studies.

The current version of the ACSS was tested in 228 women visiting urologist’s office for different reasons. The Uzbek, as well as the Russian versions, are clear and understandable for women of any age, educational level, and employment status. Our study demonstrated high reliability and validity of the questionnaire. The items of ACSS have excellent internal consistency and validity.

The results of the present study, including only the 228 women to whom the current ACSS was applied, did not show clinically relevant diagnostic differences to the study published earlier [19], which included an additional 58 women, to whom the precursor questionnaire, USQOLAT, was administered (Table 1).

Health-related quality of life is gaining increasing importance, combining characteristics of physical, psychological, emotional, and social human functioning based on subjective perception [33,34,35]. The assessment of the quality of life is usually performed by patient self-reporting and may be much more useful even if it mismatches the physician’s view. Although standard medical and biological parameters are often basic criteria of treatment efficacy, they cannot reflect the patient’s feeling of well-being and performance of everyday activities. Therefore, indeed the self-assessment may be the most informative index of the patient’s status of health in many cases [36]. In this context, symptom scores and questionnaires are valuable tools. In urology, international validated questionnaires such as the IPSS (International Prostate Symptom Score) and the NIH-CPSI (National Institutes of Health Chronic Prostatitis Symptom Index) are broadly used for various conditions [37,38].

Until recently, there was no generally accepted questionnaire for AC available for combined assessment of severity of symptoms and impact on quality of life. Our study aimed to develop a highly sensitive and specific as well as simple patient self-reporting questionnaire assessing the symptoms of AC and their impact on quality of life, differentiating AC from other urogynecological disorders with similar symptomatology, and assessing treatment efficacy.

In previous studies on UTIs, numerous different diaries, symptoms questionnaires and scales for separately assessing “bladder symptoms” and health-related quality of life were used separately [4,15,16,27,28,39,40,41]. Some researchers assessed general conditions and wellbeing of patients rather than urinary symptoms [17,29]. Others have used “self-made” questionnaires or algorithmic schemes for assessing symptoms in patients with pre-diagnosed AC before and after treatment [12,13,39]. The symptom score used in the study of Bleidorn et al. [27] contained questions regarding only three key symptoms of AC (dysuria, frequency, and lower abdominal pain), while the ACSS contains also questions about other key symptoms of AC (urgency, incomplete bladder emptying and hematuria). However, the ACSS is not the first questionnaire in the area of UTIs. Earlier, two other questionnaires—the UTI Symptoms Assessment (UTISA) and the Activity Impairment Assessment (AIA)—were published [15,16]. While the UTISA evaluates the severity of lower UTI, the AIA investigates the impact on impaired activity. Unfortunately, important statistical information like sensitivity, specificity, responsiveness and discriminative abilities have not been reported for both. The ACSS was developed and validated considering these parameters. It also contains questions to differentiate AC from other commonly community-acquired urological and gynecological diseases (“Differential” domain) and items for conditions that may affect therapy (“Additional” domain) and therefore requiring broader and more thorough investigation. These additional items may add more accuracy and be useful for epidemiological surveys.

The discriminative ability of “Typical” domain of the questionnaire may allow easier distinguishment of the patients with AC from those without it. During our analysis, we have found statistically significant correlations between scores of the questionnaire and results of urinalyses. High levels of sensitivity and specificity in combination with excellent discriminative ability and responsiveness make this questionnaire very useful for diagnosing AC in women and assessment of treatment efficacy. The ACSS also evaluates the severity of symptoms and their influence on the quality of life and may assist to differentiate AC from other urological disorders with the help of the “Differential” domain. Complicating and affecting factors may be revealed by the analysis of the “Additional” domain. A special domain assessing dynamics evaluates treatment efficacy. These distinctive features are the main advantages of the ACSS compared to other existing UTI symptom scores [12,13,15,16,39].

Our study has limitations that should be acknowledged. First, the questionnaire was limited to Uzbek- and Russian-speaking women. Second, the study was performed in a single center, and only 48% of all patients with AC returned for a follow-up visit to test responsiveness. Nevertheless, the reevaluation of the questionnaire was performed in a large number of patients and controls. Results of the validation of the ACSS in German and UK English languages were published recently [42,43] and is planned or ongoing in other languages.

## 7. Conclusions

The Uzbek and the Russian ACSS was reevaluated—excluding 58 patients having filled in also the precursor version, USQOLAT—to assess the severity of symptoms in women with AC and their impact on quality of life as well as to differentiate from other urogenital disorders with the possibility to monitor treatment efficacy. The ACSS questionnaire was validated in 228 women with and without AC and demonstrated good values of reliability, validity, predictive ability, and responsiveness. This facilitates its use in the primary healthcare setting as well as in clinical studies. For a broader use validation of the ACSS in other languages will be necessary.

## 8. Patents

### Copyright and Translations of the ACSS in Other Languages

The ACSS is copyrighted by the Certificate of Deposit of Intellectual Property in Fundamental Library of Academy of Sciences of the Republic of Uzbekistan, Tashkent (Registration number 2463; 26 August 2015) and the Certificate of the International Online Copyright Office, European Depository, Berlin, Germany (Nr. EU-01-000764; 21 October 2015). The Rightholders are Jakhongir Fatikhovich Alidjanov (Uzbekistan), Ozoda Takhirovna Alidjanova (Uzbekistan), Adrian Martin Erich Pilatz (Germany), Kurt Guenther Naber (Germany), Florian Martin Erich Wagenlehner (Germany).

http://avtor-web.com/index.php?option=com_desposition&task=display_desp_det&id=2612&lang=ru  (assessed on 13 January 2018);

http://interoco.com/all-materials/work-of-science/1013-1951954939.html (assessed on 13 January 2018)

The e-USQOLAT is copyrighted by the Authorship Certificate of the International Online Copyright Office, European Depository, Berlin, Germany (Nr. EC-01-001179; 18 May 2017) 19.

http://inter.interoco.com/copyright-depository/computer-programs/1438-2017-05-18-10-59-16.html?path=computer-programs (assessed on 13 January 2018).

Translations of the ACSS in other languages are available on the website: http://www.acss.world/downloads.html.

## Figures and Tables

**Table 1:** Changes in the predictive ability of the ACSS including 228 women (107 with AC and 121 without AC) depending on the summary scores obtained in the “Typical” domain at visit 1. The most appropriate discriminative summary score for the “Typical” domain was equal to 6.

Score ≥	TP Result	TN Result	FP Result	FN Result	Sensitivity	Specificity	Pos. Predictive Value	Neg. Predictive Value	Pos. Likelihood Ratio	Neg. Likelihood Ratio	Relative Risk	Risk Difference	Diagnostic Odds Ratio	Diagnostic Effectiveness
0	107	0	121	0	100.0%	0.0%	46.9%	NA	1.0	NA	NA	NA	NA	46.9%
1	107	34	87	0	100.0%	28.1%	55.2%	100.0%	1.4	0.0	NA	55.2%	NA	61.8%
2	107	79	42	0	100.0%	65.3%	71.8%	100.0%	2.9	0.0	NA	71.8%	NA	81.6%
3	106	85	36	1	99.1%	70.2%	74.6%	98.8%	3.3	0.0	64.2	73.5%	250.3	83.8%
4	104	96	25	3	97.2%	79.3%	80.6%	97.0%	4.7	0.0	26.6	77.6%	133.1	87.7%
5	100	101	20	7	93.5%	83.5%	83.3%	93.5%	5.7	0.1	12.9	76.9%	72.1	88.2%
6	99	108	13	8	92.5%	89.3%	88.4%	93.1%	8.6	0.1	12.8	81.5%	102.8	90.8%
7	96	110	11	11	89.7%	90.9%	89.7%	90.9%	9.9	0.1	9.9	80.6%	87.3	90.4%
8	86	113	8	21	80.4%	93.4%	91.5%	84.3%	12.2	0.2	5.8	75.8%	57.8	87.3%
9	78	116	5	29	72.9%	95.9%	94.0%	80.0%	17.6	0.3	4.7	74.0%	62.4	85.1%
10	65	119	2	42	60.7%	98.3%	97.0%	73.9%	36.8	0.4	3.7	70.9%	92.1	80.7%
11	53	120	1	54	49.5%	99.2%	98.1%	69.0%	59.9	0.5	3.2	67.1%	117.8	75.9%
12	42	121	0	65	39.3%	100.0%	100.0%	65.1%	NA	0.6	2.9	65.1%	NA	71.5%
13	30	121	0	77	28.0%	100.0%	100.0%	61.1%	NA	0.7	2.6	61.1%	NA	66.2%
14	19	121	0	88	17.8%	100.0%	100.0%	57.9%	NA	0.8	2.4	57.9%	NA	61.4%
15	16	121	0	91	15.0%	100.0%	100.0%	57.1%	NA	0.9	2.3	57.1%	NA	60.1%
16	8	121	0	99	7.5%	100.0%	100.0%	55.0%	NA	0.9	2.2	55.0%	NA	56.6%
17	5	121	0	102	4.7%	100.0%	100.0%	54.3%	NA	1.0	2.2	54.3%	NA	55.3%
18	3	121	0	104	2.8%	100.0%	100.0%	53.8%	NA	1.0	2.2	53.8%	NA	54.4%

TP = true-positive; TN = true-negative; FP = false-positive; FN = false-negative.

**Table 2:** Predictive ability of the current ACSS including 228 women (107 with AC and 121 without AC) using for diagnostics of AC a summary score of 6 or higher obtained in the “Typical” domain at visit 1 as compared with the study published earlier [19] including in addition 58 women (total 286), in whom the precursor questionnaire, USQOLAT, was applied.

Questionaire	ACSS	ACSS + USQOLAT
Women total (*n*)	228	286
Women with AC	107	139
Women without AC	121	147
Sensitivity	92.5%	93.5%
Specificity	89.3%	89.8%
True-positive (*n*, %)	99 (92.5%)	138 (99.2%)
True-negative (*n*, %)	108 (89.3%)	132 (89.8%)
False-positive (*n*, %)	13 (10.7%)	15 (10.2%)
False-negative (*n*, %)	8 (7.5%)	9 (6.5%)
Positive predictive value	88.4%	89.7%
Negative predictive value	93.1%	93.6%
Positive likelihood ratio	8.6	9.2
Negative likelihood ratio	0.1	0.1
Relative risk	12.8	14.0
Risk difference	81.5%	83.3%
Diagnostic Odds ratio	102.8	127.1
Diagnostic effectiveness	90.8%	91.6%

**Table 3:** Estimation of risks depending on “cut-off” value in “Typical” domain of ACSS at visit 1.

	‘Typical’ Score ≥ 6	‘Typical’ Score < 6
Number of subjects	112	116
Risk of having AC	0.88	0.07
Risk of not having AC	0.12	0.93
Odds of having AC	7.62	0.07
Odds against having AC	0.13	13.5
Absolute risk difference	81.5%	
Odds ratio	102.81	

**Table 4:** Differences in scores of the ACSS between patients with AC (*n* = 107) and controls without AC (*n* = 121) at visit 1.

Item/Domain	Mann-Whitney U	Patients (*n* = 107)		Controls (*n* = 121)		*p*-Value
		Mean	SD	Mean	SD	
Frequency	1720.50	2.05	0.85	0.71	0.68	0.000
Urgency	1364.50	2.07	0.95	0.28	0.70	0.000
Painful urination	1066.50	2.09	0.91	0.24	0.66	0.000
Incomplete bladder emptying	1329.50	1.87	0.81	0.36	0.74	0.000
Suprapubic discomfort	1761.50	1.77	0.90	0.39	0.78	0.000
Hematuria	4155.00	0.66	0.90	0.09	0.37	0.000
**Total “Typical” score**	458.50	10.50	3.49	2.07	2.55	0.000
Flank pain	6097.00	1.32	1.01	1.40	0.97	0.428
Vaginal discharge	5539.00	0.49	0.79	0.26	0.60	0.013
Urethral discharge	5573.00	0.27	0.61	0.09	0.39	0.002
Feeling of chill/fever	5774.50	0.27	0.62	0.14	0.49	0.018
Hyperthermia (measured) *	5883.00	0.27 ^a^	0.59	0.17 ^b^	0.53	0.058
**Total “Differential” score**	5630.50	2.35	1.84	1.90	1.51	0.082
General dyscomfort	2923.00	2.06	0.66	1.18	0.86	0.000
Impairment of everyday activity	2541.00	1.87	0.67	0.91	0.84	0.000
Impairment of social activity	2863.50	1.75	0.75	0.84	0.83	0.000
**Total “QoL” score**	2396.50	5.67	1.80	2.93	2.34	0.000

* Hyperthermia (measured): ^a^ *n* = 90; ^b^ *n* = 94.

**Table 5:** The number of patients with AC (*n* = 107) with symptoms of severity “moderate” (score 2) and “severe” (score 3) in “Typical” and “Differential” domain of the ACSS at visit 1.

Domain	Symptom	‘Moderate’	‘Severe’	Total	Percentage
Typical	Urgency	46	40	86	80.4%
	Painful urination	43	41	84	78.5%
	Frequency	45	36	81	75.7%
	Incomplete bladder emptying	47	25	72	67.3%
	Suprapubic discomfort	40	25	65	60.7%
	Hematuria	16	15	31	29.0%
Differential	Flank pain	32	15	47	43.9%
	Vaginal discharge	11	3	14	13.1%
	Feeling of chill/fever	4	2	6	5.6%
	Hyperthermia	5	1	6	5.6%
	Urethral discharge	3	2	5	4.7%

**Table 6:** Distribution of scores in Typical Symptoms of ACSS in women without AC (Controls) and in those with AC (Patients).

Symptom/Severity	Controls (*n* = 121)	Patients (*n* = 107)
**Frequency**		
No (4 or less times per day)	41.32%	4.67%
Yes, mild (5–6 times/day)	46.28%	19.63%
Yes, moderate (7–8 times/day)	12.40%	42.06%
Yes, severe (9–10 or more times/day)	0.00%	33.64%
**Urgency**		
No	81.82%	11.21%
Yes, mild	12.40%	8.41%
Yes, moderate	1.65%	42.99%
Yes, severe	4.13%	37.38%
**Painful urination**		
No	85.95%	7.48%
Yes, mild	6.61%	14.02%
Yes, moderate	4.96%	40.19%
Yes, severe	2.48%	38.32%
**Incomplete bladder emptying**		
No	77.69%	3.74%
Yes, mild	11.57%	28.97%
Yes, moderate	8.26%	43.93%
Yes, severe	2.48%	23.36%
**Suprapubic pain**		
No	76.86%	7.48%
Yes, mild	9.92%	31.78%
Yes, moderate	10.74%	37.38%
Yes, severe	2.48%	23.36%
**Visible blood in the urine**		
No	93.39%	57.94%
Yes, mild	4.13%	22.43%
Yes, moderate	2.48%	14.95%
Yes, severe	0.00%	4.67%

**Table 7:** Differences (Mann-Whitney test) in total “Typical” scores of the ACSS between patients with AUC (*n* = 107) and controls without AUC (*n* = 121), substratified in pregnant women, women 50 years of age and younger, and women 51 years of age and older. N: number; SD: standard deviation; CI: confidence interval.

	Patients with AUC				Controls without AUC				*p*-Value *
		“Typical” Scores				“Typical “Scores			
Respondents	*N*	mean	SD	95% CI	*N*	mean	SD	95% CI	
Pregnant women	21	9.29	4.11	7.41–11.16	20	2.55	3.03	1.13–3.97	4.00 × 10^−6^
≤50 years, not pregnant	79	10.73	3.32	9.99–11.48	87	2.05	2.45	1.52–2.57	2.51 × 10^−26^
≥51 years	7	11.57	2.82	8.96–14.18	14	1.50	2.50	0.05–2.95	3.40 × 10^−05^
Total	107	10.50	3.49	9.84–11.17	121	2.07	2.55	1.61–2.53	4.07 × 10^−34^

* *p* values in the table are given in scientific (exponential) notation and are lower than 0.0001 for all cases.

**Table 8:** Differences in scores between first and follow-up visit (*n* = 48).

Scores in Different Domains of the ACSS	First Visit		Subsequent Visit		Mean Difference ^a^	*p*-Value ^b^
	Mean	SD	Mean	SD		
Total “Typical” score	10.8	3.2	2.5	3.2	8.3	<0.0001
Total “Differential” score	2.2 ^a^	1.6	0.8 ^a^	1.2	1.4	<0.0001
Total “QoL” score	5.7	1.7	1.5	2.2	4.2	<0.0001

^a^ Based on the sum of scores of 46 patients with non-missing values. ^b^ *p*-values are generated by Wilcoxon Signed Rank Test.

## Supplementary Material

Figure S1: Updated Uzbek (Cyrillic) version of the Acute Cystitis Symptom Score (ACSS) for the first visit. Website: http://www.acss.world/ACSS_UZ_C.pdf (assessed on 13 January 2018). Figure S2: Updated Russian version of the Acute Cystitis Symptom Score (ACSS) for the first visit. Website: http://www.acss.world/ACSS_RU.pdf (assessed on 13 January 2018).
